# Exploring the Neural Correlates of Flow Experience with Multifaceted Tasks and a Single-Channel Prefrontal EEG Recording

**DOI:** 10.3390/s24061894

**Published:** 2024-03-15

**Authors:** Yuqi Hang, Buyanzaya Unenbat, Shiyun Tang, Fei Wang, Bingxin Lin, Dan Zhang

**Affiliations:** 1 Department of Psychology, School of Social Sciences, Tsinghua University, Beijing 100084, China; yh2072@nyu.edu (Y.H.); wf3126@mail.tsinghua.edu.cn (F.W.); 2 RDFZ Xishan School, Beijing 100091, China

**Keywords:** flow experience, single-channel EEG, multifaceted tasks

## Abstract

Flow experience, characterized by deep immersion and complete engagement in a task, is highly recognized for its positive psychological impacts. However, previous studies have been restricted to using a single type of task, and the exploration of its neural correlates has been limited. This study aimed to explore the neural correlates of flow experience with the employment of multifaceted flow-induction tasks. Six tasks spanning mindfulness, artistic tasks, free recall, and varying levels of Tetris complexity (easy, flow, and hard conditions) were employed to have relatively complete coverage of the known flow-induction tasks for a better induction of individualized flow experience. Twenty-eight participants were recruited to perform these six tasks with a single-channel prefrontal EEG recording. Significant positive correlations were observed between the subjective flow scores of the individual’s best-flow-experience task and the EEG activities at the delta, gamma, and theta bands, peaking at latencies around 2 min after task onset. The outcomes of regression analysis yield a maximum R^2^ of 0.163. Our findings report the EEG correlates of flow experience in naturalistic settings and highlight the potential of portable and unobtrusive EEG technology for an objective measurement of flow experience.

## 1. Introduction

Flow experience, a subjective state of deep immersion and engagement in a task [1], has been extensively studied, underscoring its significant impact on individual motivation, skills, and performance across various domains. Notably, performance enhancements associated with the flow state have been documented in diverse fields such as artistic expression [2], sports [3], music [4], and education [5]. Within laboratory environments, numerous paradigms have been employed to induce flow, including but not limited to arithmetic tasks [6], sports or physical activities [7,8], video games [9,10], arts [2,11,12], mindfulness or meditation practices [13,14], free recall exercises [15,16], and dual-task paradigms [17,18]. Specifically, the Tetris game is a widely used paradigm to induce flow, known for its simplicity, adjustable difficulty, instant feedback, clear goals, and quantifiable controls [19]. Keller et al. [9] once employed the Tetris game to study flow characteristics under various difficulty levels tailored to individual skills. Mindfulness is closely associated with the state of flow, and multiple studies have shown that mindfulness training can enhance the flow experience [13,14]. Drawing is a relatively accessible way to initiate the flow state and is commonly used in art therapy to facilitate flow [2]. Additionally, coloring games have also been shown to generate flow experiences [11,12]. Recall is also a commonly used method for inducing flow, where participants can exhibit similar emotional responses by recalling past experiences [15,16].

However, traditional methods of assessing flow primarily rely on self-report scales or questionnaires [20,21,22]. These methods are subject to limitations due to their interruptive nature and reliance on personal perceptions, which can be influenced by mood [23], memory [24], and cognitive biases [25], thus constraining the understanding and further application of flow mechanisms. The advent of neuroscience-based measurement techniques for flow could offer promising breakthroughs [26]. Utilizing neuroscientific tools such as electroencephalography (EEG) and functional near-infrared spectroscopy (fNIRS) [26], it is now possible to measure an individual’s neural activities continuously and non-intrusively throughout the immersion in the flow experience. This approach enables an exploration of the neural underpinnings of flow and the development of neuroengineering applications based on flow dynamics.

Investigating the neural correlates of psychological flow is essential for developing neuroscience-based measurement techniques for flow. In the field of positive psychology, extensive research has been conducted, utilizing techniques such as EEG [27], fNIRS [28], and a multi-channel electrodermal measurement device [29] to explore the physiological correlates of positive emotions. While the neuroscience research on flow is still emerging, several studies have already begun to employ various approaches in an attempt to explore its neural basis [26]. Specifically, Ulrich et al. [30] employed functional magnetic resonance imaging combined with a mental arithmetic task, revealing reduced activity in the medial prefrontal cortex during flow states compared with overload and boredom conditions. Utilizing the same arithmetic task, an EEG study found higher theta power in frontal electrodes during flow and overload conditions, and increased alpha power during flow compared to boredom [6]. Furthermore, certain studies have employed fNIRS in conjunction with Tetris tasks, revealing increased activity in the prefrontal cortex during the flow condition in contrast to the boredom condition [31,32]. Despite the use of different techniques, these studies seem to indicate that the prefrontal cortex is a pivotal brain region in facilitating the flow experience [33,34]. However, prior research predominantly relied on research-grade equipment, which imposes limitations on the practical application of flow studies.

Recent advancements in neuroscience measurement technologies, particularly the development of portable, lightweight, and low-cost EEG technology, are increasingly being applied across various fields, facilitating widespread use and accessibility. In the field of personality psychology, EEG or heart rate data collected by wearable devices could be potentially applied to predict big-five personality traits in daily life [29,35]. In psychiatry, portable EEG devices, specifically single-channel prefrontal EEG, have been utilized for neurofeedback training in children with ADHD, focusing on individual beta rhythm [36]. Single-channel EEG could also be used to assess the maturation of children’s attention control through the task-related frontal EEG theta/beta ratio [37]. In the realm of the arts, mobile EEG devices have been employed to objectively measure aesthetic preferences in dance appreciation studies [38]. In sports, portable EEG applications may offer an efficient and valid approach for predicting anxiety levels in soccer players, with a focus on the prefrontal region [39]. Furthermore, in educational research, inter-brain coupling analysis [40] combined with single-channel prefrontal EEG has been used to reflect disciplinary differences between subjects like Chinese and Mathematics in real-world classroom learning [41]. The congruence between the neural region targeted by single-electrode prefrontal EEG, a leading-edge technology within the domain of portable EEG systems, and the critical neural substrates implicated in flow experiences [33,34] underscores the potential of single-channel prefrontal EEG as an invaluable instrument for propelling application-focused research on psychological flow. Specifically, this portable EEG device has made it feasible to study the neural correlates of flow states in real-world settings outside the confines of traditional laboratory environments. By enabling the collection of EEG data in naturalistic settings, researchers can observe the dynamics of flow states during various activities. Also, portable EEG that can detect flow states could be easily applied to potential neurofeedback training in the future that facilitates the occurrence of psychological flow, helping people achieve optimal performance in multiple settings.

The application of portable devices allows for a more diverse exploration of tasks in psychological flow research. Traditionally, laboratory neuroscience studies have often focused on a single-task paradigm for flow induction [30,42,43]. However, relying solely on a single task to induce flow presents inherent limitations, as responses to different paradigms vary due to individual, interindividual, contextual, and cultural factors [44]. Certain paradigms may be more effective than others in inducing flow in specific individuals. To address this variability and enhance the characterization of individualized flow states, it is proposed to employ multiple tasks to induce flow states in each participant, thanks to the portability of EEG devices that makes measuring flow in multiple paradigms much more convenient. This approach allows for the identification and utilization of the paradigm that induces the strongest flow experience for each individual. By doing so, this study aims to capture a more personalized representation of an individual’s flow state in our experiments, thus providing a more comprehensive understanding of the flow experience.

The present study aims to explore the neural representation of flow based on prefrontal single-channel EEG. A cohort of twenty-eight participants was outfitted with portable EEG devices as they engaged in six distinct flow-induced activities: mindfulness, art-related tasks, free recall, and varying difficulty levels of Tetris (easy, flow, and hard conditions). Drawing from prior research, observing individual variations in tasks for inducing flow is anticipated. Additionally, significant correlations are expected to emerge between the power of specific EEG bands in the prefrontal cortex and the subjectively reported psychological flow experienced during certain time segments. By employing a range of regression models, it is feasible to utilize the five key EEG frequency bands—delta, theta, alpha, beta, and gamma—as predictors for subjective flow experiences.

## 2. Materials and Methods

### 2.1. Participants

This study engaged 28 participants (14 males, 14 females; mean age: 20 ± 2.7 years, ranging from 18 to 28 years), comprising 9 individuals from China and 19 from Mongolia. 15 participants were from art-related majors. They reported no neurological or psychological disorders, to be right-handed, and to have normal or corrected-to-normal vision. Each participant received a remuneration of 100 RMB per hour. The research received approval from the local ethics committee of Tsinghua University. Informed consent was obtained from all participants prior to the experiments.

### 2.2. Tasks

There were one resting state condition and six different experimental task conditions that induced flow states in this study, including three different difficulty levels of Tetris games (easy, flow, and hard conditions), each lasting around 10 min, as well as three other tasks, which were mindfulness, art activities, and free-recall, each lasting around 5 min.

In the Tetris game task, participants played a popular game called Tetris, where falling pieces of a great variety of geometrical shapes were required to get arranged to form lines. Three game speeds were applied: slow, medium, and fast, for easy, flow, and hard conditions, respectively, through following Harmat et al.’s [10] design that matched difficulty to skill. Specifically, an adaptive 15-min session determined the baseline speed (Sbalance) for each participant. During the adaptive phase, participants could use the four directional keys on the keyboard (the up key for rotating the blocks, the down key for fast downward movement, the left key for leftward movement, and the right key for rightward movement) to play the game. At the end of the adaptive phase, the game speed, also the falling speed of the geometrical shapes, would converge to a speed (Sbalance) that matched the participants’ gaming performance through the abilities of controlling keys to form lines, which served as the baseline for subsequent phases. The speed of the flow condition (Sflow) was equal to the speed of the balance (Sbalance), the speed of the hard condition (Shard) was 2.5 times the speed of the balance (Sbalance), while the speed of the easy condition (Seasy) was 0.4 times the speed of the balance (Sbalance). The order of these three conditions was randomized for each individual.

In the mindfulness task, participants were provided with an audio recording which offered explicit directives on the nuances of controlled breathing and the subtleties of achieving an optimal meditative stance. For instance, the audio described the process of diaphragmatic breathing, instructing participants to inhale deeply through the nose, allowing the abdomen to rise, and to exhale slowly through the mouth, letting the body relax progressively with each breath. Additionally, the guide included instructions for aligning the spine, positioning the hands gently on the laps, and achieving a state of relaxed alertness that is central to effective meditation. As they listened, the participants were encouraged to immerse themselves fully in the experience, synchronizing their breathing patterns with the rhythmic cues provided and adjusting their postures in real-time as per the guidance.

In the art task, the task was divided into drawing and coloring. This setup took into consideration the participants’ different backgrounds, where those from art-related majors could enter the state of flow through drawing, while participants without an art background might find it challenging to achieve flow due to personal drawing abilities. Therefore, for non-art professionals, a user-friendly coloring game was chosen as an alternative method to induce flow. In the drawing task, each participant was provided with an A4 white paper and a 2B pencil, and he/she was instructed to create freely. In the coloring task, participants used an iPad Air (4th generation) to color a moderately challenging picture in the “Flower Coloring” app.

In the free-recall task, participants were prompted to delve into their memories and retrieve specific instances where they experienced a state of flow, following detailed verbal instructions. The facilitator guided them through a reflective process, encouraging them to think back to times when they were completely absorbed in an activity, to the extent that they lost track of time and were solely focused on the task at hand. For example, they were asked to recount moments when they were engaged in a creative endeavor, such as painting or writing, where ideas seemed to come effortlessly. Alternatively, they might have been prompted to reflect on occasions involving physical activities, like playing a musical instrument or participating in a sport, where their movements and actions flowed seamlessly from one to the next.

At last, to establish a baseline neural state for each participant, a resting state session was employed, following previous studies [45]. This involved instructing participants to remain still for 5 min and refrain from engaging in any specific tasks.

### 2.3. Procedure

**Figure 1:**
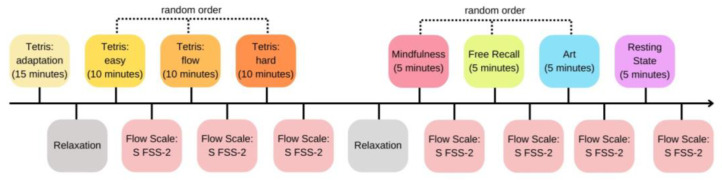
The experimental procedure for the multifaceted flow tasks. ‘S FSS-2’ refers to the Chinese edition of the Short Flow State Scale-2.

The experiment consisted of one adaptive condition, six task conditions, two relaxation conditions, and one resting-state condition. Participants first filled in subject information before conducting an adaptive condition. Afterward, there was a relaxation condition, followed by three task conditions of different difficulty levels of Tetris games (easy, flow, hard) in random order. After each Tetris game condition, participants were required to report their subjective flow states through a flow questionnaire. When all levels of Tetris game conditions and corresponding questionnaire data collection were completed, another relaxation condition followed. After relaxation, participants were required to conduct mindfulness, art activities, and free-recall conditions in random order, and after each, they completed the flow questionnaire. Finally, each participant’s resting-state was recorded. Six self-reported scores of flow states for six different tasks were obtained. In terms of the flow questionnaire, it was the Chinese edition of the Short Flow State Scale-2 (S FSS-2) [46], in which nine items were rated on a five-point Likert-scale ranging from 1 (“I completely disagree”) to 5 (“I completely agree”). The workflow is depicted in Figure 1.

### 2.4. EEG Recordings

Participants’ brain signals were recorded at Fpz over the forehead using a single-channel headband (CUBand, CUSoft, Beijing, China) with the NeuroSky EEG biosensor supported by ThinkGear technology (NeuroSky, San Jose, CA, USA). The reference electrode was placed on the left ear lobe with a ground at Fp1. ThinkGear, including the sensor, the contact and reference points, and the onboard chip that processes all of the data, represents NeuroSky’s proprietary technology that employs a single dry sensor for capturing, amplifying, purifying, and interpreting EEG signals and neural oscillations. When integrated with NeuroSky’s exclusive eSense™ algorithms, the technology equips a headset with the capability to assess the user’s mental state. In terms of data preprocessing, the signal fidelity of the NeuroSky EEG biosensor employed in the context of the current study has been corroborated through prior validation [47]. The NeuroSky EEG biosensor utilized ThinkGear technology during the data collection phase to eliminate noises, disturbances from additional electrical devices, ocular motion-related artifacts, and AC interference. Also, recognizing that a degree of noise is inherent in the standard operation of ThinkGear, NeuroSky has developed both its filtering technology and the eSense™ algorithm to identify, rectify, adjust for, and withstand various forms of noise unrelated to EEG signals. There is an integer value called Poor_Signal_Quality that describes how poor the signal measured by ThinkGear is, ranging from 0 to 255; the higher number indicates more detected noise. ASIC EEG Power at different frequency bands was calculated by the ThinkGear chips within the headband for every second. The extracted features are Delta (1~3 Hz), Theta (4~7 Hz), Alpha (8~12 Hz), Beta (13~30 Hz), and Gamma (31~50 Hz). The EEG data were measured in terms of ASIC EEG power, representing the relative amplitudes of individual EEG bands without a conventional unit [48].

### 2.5. Data Analysis

In this study, 28 participants engaged in six activities specifically designed to elicit flow states, subsequently rating their subjective flow experiences for each activity. Initially, a validation analysis was performed to examine variations in subjective scores under three Tetris gameplay conditions: easy, flow, and hard, to see whether the objective difficulty level of Tetris can truly reflect subjective flow experiences. Given the interrelated nature of the samples and the non-normal data distribution in the hard Tetris condition, a nonparametric Friedman test was employed, supplemented by a post hoc Nemenyi test, for the analysis. To account for multiple comparisons, False Discovery Rate (FDR) corrections were applied using the Benjamini–Hochberg procedure.

Furthermore, to identify the condition that most frequently elicited the highest flow scores among participants and, most importantly, to understand the individual differences in tasks that induce the strongest flow experience, each participant’s flow scores across the six activities were ranked. A higher ranking indicated a stronger flow experience in that particular task. For example, if a participant’s highest flow score was in the art task, this was assigned a rank of 1. The task with the highest frequency of rank 1 scores was deemed the most effective at inducing flow at the group level. Histograms depicting the distribution of flow scores across all ranks were also generated, enabling an analysis of the internal dynamics and interrelations of these distributions and highlighting the variation in flow scores across different ranks.

Prior to the formal EEG analysis, we first tested the reliability of the recorded EEG data by comparing the EEGs during the mindfulness state and those during the resting state, as practiced in previous studies [49]. The first 240 s of EEG data were utilized to ensure that the participants were in the expected state. The non-parametric Wilcoxon signed-rank test was then applied to compare the ASIC EEG power in the alpha band during mindfulness versus resting states, as the ASIC EEG power data did not follow the normal distribution. Following previous studies [49], a larger alpha power is expected for the mindfulness state as compared to the resting state.

In the formal EEG analysis, individual differences and the temporal dynamics of flow experiences were considered. The EEG data were averaged every 30 s across the delta, theta, alpha, beta, and gamma frequency bands under all experimental conditions, following baseline correction by subtracting the averaged EEG power during resting conditions from EEG power under experimental conditions. The focus was on each participant’s most intense flow experience and its associated brain–behavior relationships. The flow experience with the highest flow score and corresponding neural data was selected for each participant to perform a correlation analysis. For each frequency band, the Pearson correlation between ASIC EEG power and the subjective flow scores was calculated for each of the eight time segments. A significant correlation in this context may suggest that neural signals effectively represent psychological flow within specific frequency bands and time segments.

In the final phase of our study, a leave-one-subject-out cross-validation regression analysis was performed to evaluate the predictive capability of EEG power across all the five frequency bands combined and each of the five frequency bands (delta, theta, alpha, beta, gamma), respectively, for estimating the subjective flow score. In the regression models, the data were partitioned into two distinct sets: a training set and a test set. The training set was utilized to develop and train the model, allowing it to learn the underlying patterns and relationships within the data. The test set, comprising data points not used during the training phase, was employed to evaluate the model’s performance. This approach ensured that our model’s predictive accuracy was assessed on previously unseen data, thereby providing a more reliable indication of its generalizability and effectiveness in real-world scenarios. For each participant, six flow tasks were conducted, and the task with the highest flow score was identified as the best task for each individual. Across eight time segments, EEG power of the combination of five frequency bands and each of the five frequency bands during the best tasks of each of the 27 participants was selected as features for the training set (X_train), while the subjective flow scores of these best tasks were chosen as labels for the training set (y_train). For the one left-out participant, EEG power during their best task was selected as features for the test set (X_test), and the subjective flow score of this best task was chosen as the label for the test set (y_test). Before training the machine learning models, the feature sets for both the training set (X_train) and the test set (X_test) underwent standardization. This process involved first fitting and transforming the training set features with a standard scaler, thereby normalizing them based on the training set’s own distribution. The parameters derived from the training set’s standardization process were then applied to the test set features. Subsequently, four different machine learning models were utilized to train and validate the data: a linear regression model, a random forest regression with n_estimators set to 10, a KNN regression with n_neighbors set to 5, and an Elastic Net regression with alpha set at 0.5 and l1_ratio at 0.5. Given that there are 28 participants, each round of cross-validation was executed 28 times, each time generating a model’s predicted value. Finally, the coefficient of determination (R^2^) value was computed for each validation round, based on the 28 pairs of model predictions and actual observations, calculated according to Formula (1) [50] as below.

(1) $$R^{2}=1-\frac{\sum {\left(y_{i}-\hat{y}\right)}^{2}}{\sum {\left(y_{i}-\bar{y}\right)}^{2}}$$

Formula (1): R^2^. $y_{i}$ represents the ith observation of the target variable, $\hat{y}$ represents the predicted value of the target variable, and $\bar{y}$ represents the mean of observed values of the target variable.

Accordingly, R^2^ was computed for all eight time segments for all frequency bands of interests and all four types of machine learning models.

## 3. Results

### 3.1. Individuality of Tasks That Induce the Strongest Flow Experience

**Figure 2:**
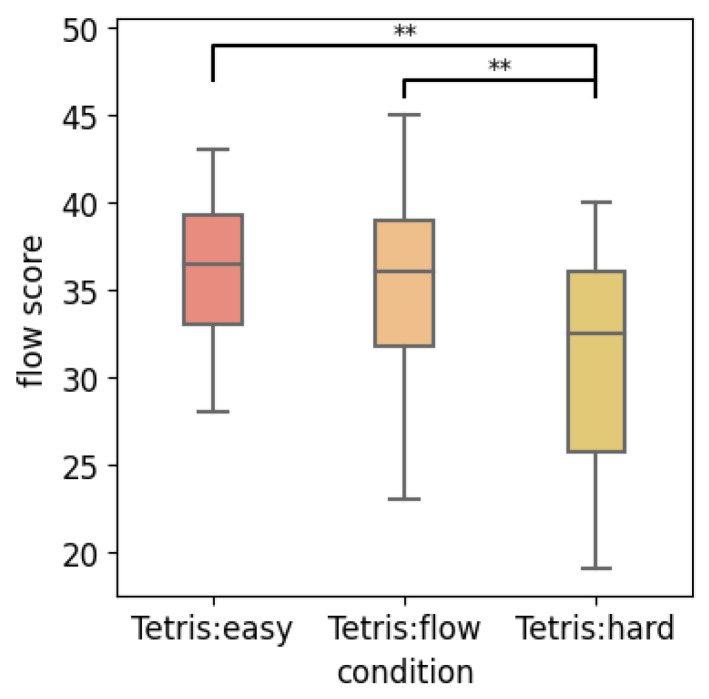
Comparison of subjective flow scores among three conditions of Tetris (**: *p* < 0.01).

To investigate whether the objective difficulty level of Tetris gameplay can genuinely reflect subjective flow experiences, a comparative analysis of subjective flow scores across three different difficulty levels was conducted (Figure 2). The mean subjective flow scores for the easy, flow, and hard versions of Tetris gameplay were as follows: easy condition (*M* = 35.93, *SD* = 4.26), flow condition (*M* = 34.71, *SD* = 5.33), and hard condition (*M* = 30.96, *SD* = 6.36), as illustrated in Figure 1. The Friedman test indicated that at least one condition significantly differed from the others (*χ*^2^ = 21.981, *df* = 2, *p* < 0.001). Subsequent post hoc analyses using the Nemenyi test with FDR correction revealed significant differences between the easy and hard conditions (*adjusted p* = 0.003) and between the flow and hard conditions (*adjusted p* = 0.004). These findings suggest that participants reported lower subjective flow scores during the hard condition compared to the easy and flow conditions. This replicates previous findings to confirm the effectiveness of our study. However, subjective flow scores during the flow condition are not higher than during the easy condition. This outcome presents a deviation from previous research, indicating that the flow condition of Tetris gameplay does not necessarily induce the highest subjective flow experiences when compared to both easy and hard conditions. Therefore, results suggest that the objective difficulty level may not accurately reflect an individual’s subjective flow experience, highlighting the importance of assessing one’s best flow performance based on self-reported flow scores rather than relying solely on the objective difficulty level of Tetris gameplay.

**Figure 3:**
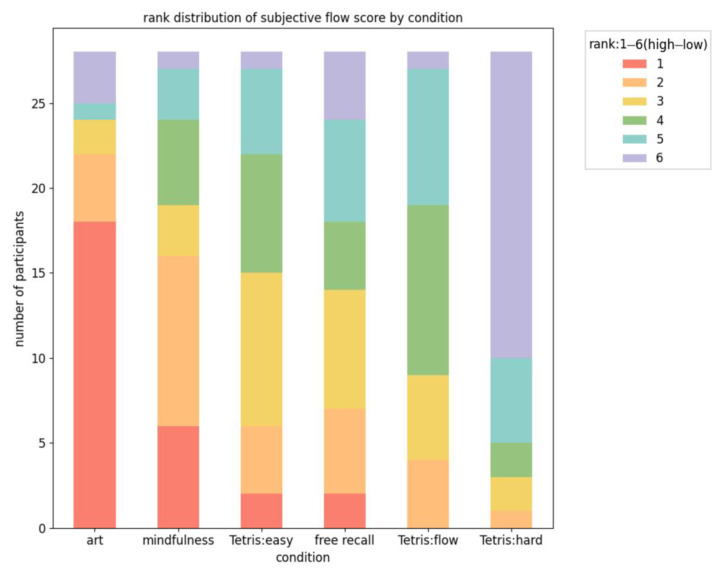
Rank distribution of subjective flow score by condition. This rank distribution shows how many times each condition was assigned a particular rank by the participants. Each color represents a different rank, and the height of the color segment shows how many participants assigned that rank to the condition.

Next, the best flow experiences across six different activities, as determined by self-reported flow ratings, were evaluated. The frequency of each activity receiving a specific ranking from participants was quantified, with higher rankings indicating more intense flow experiences (Figure 3). Regarding activities that most effectively elicited flow (ranked first), 18 participants preferred artistic activities, six favored mindfulness, two were most engaged by an easy version of Tetris, and two found free-recall tasks the most absorbing. This suggests that while artistic activities generally have a higher propensity for inducing flow at the group level, there remains considerable individual variation in best flow experiences. In contrast, activities least likely to induce flow (ranked sixth) varied, with 18 participants struggling with Tetris in hard mode, four with free-recall tasks, and six with assorted other activities. This diversity in flow-inducing activities across different ranks underscores the significant variability in factors that influence flow intensity among individuals. Essentially, the triggers for high flow states are highly individualized, reinforcing the concept of unique flow experiences. This highlights the importance of recognizing that even the most effective tasks for inducing the strongest flow experiences differ from person to person.

**Figure 4:**
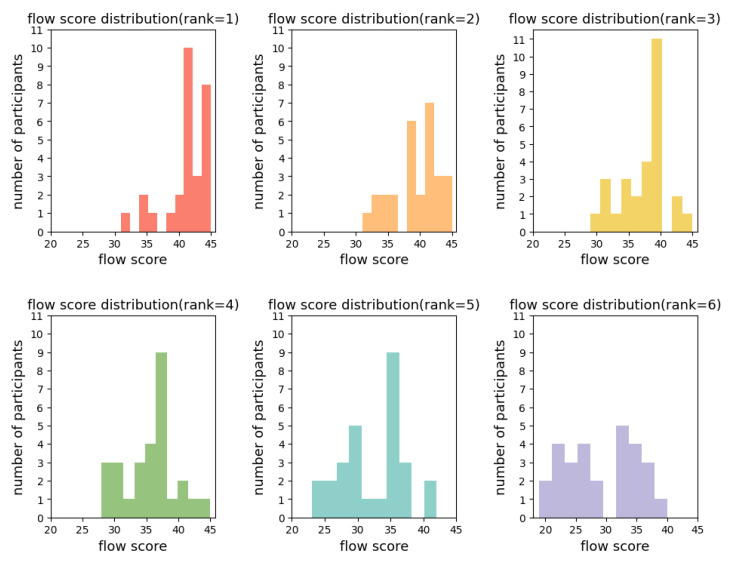
Flow score distribution by different ranks. This presents a series of six histograms, each correlating to a distinct rank, numbered from 1 to 6. The horizontal axis (*x*-axis) of each histogram delineates the flow scores, while the vertical axis (*y*-axis) quantifies the number of participants for each of these scores.

Then, histograms were used to represent the distribution of number of participants of flow scores across each specified rank (Figure 4). Flow scores across various ranks display a heterogeneous distribution, underscoring the presence of individual variability within each rank. Particularly for rank 1, there is a marked concentration of flow scores on the upper spectrum, but the distribution is not monolithic. Instead, there is a discernible spread across a variety of scores, which reveals a degree of individual variation as opposed to a singular, dominant peak at the highest score. This spread implies that although there is a general inclination towards higher flow scores within rank 1, this is not uniformly the case for all observations or individuals within that category. Consequently, this denotes a heterogeneous array of outcomes within that rank.

### 3.2. EEG Correlates of Flow Experience

**Figure 5:**
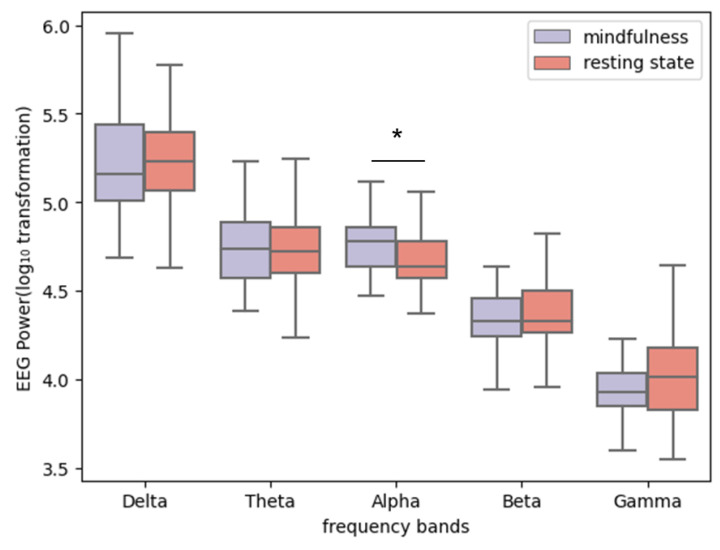
EEG power of five frequency bands between mindfulness and resting state conditions (*: *p* < 0.05).

In this research, our investigation was initiated with an analysis of data quality to validate the EEG measurements obtained. Only 0.04% of the entire dataset, equivalent to 3 s, contains slight noises (with Poor_Signal_Quality being lower than or around 50), which is regarded as tolerable. Figure 5 illustrates a discernible trend: as EEG frequency bands progress from lower (e.g., delta and theta) to higher frequencies (e.g., alpha, beta, and gamma), there is a reduction in EEG power. This unsurprising result is consistent with previous findings [48], serving as one of the indicators of the reliability of our data. Moreover, the application of the Wilcoxon signed-rank test revealed a statistically significant disparity between mindfulness and resting state conditions in the alpha band. Specifically, EEG power within the alpha band was significantly higher during mindfulness exercises compared to the resting state (*z* = −2.459, *p* = 0.013), a finding depicted in Figure 5. This observation is in line with existing literature [49], where an increase in alpha power during mindfulness practices, relative to resting states, has been consistently reported. The consistency of our findings with those of established studies, particularly in the context of using portable EEG devices, reaffirms the reliability of our data.

**Figure 6:**
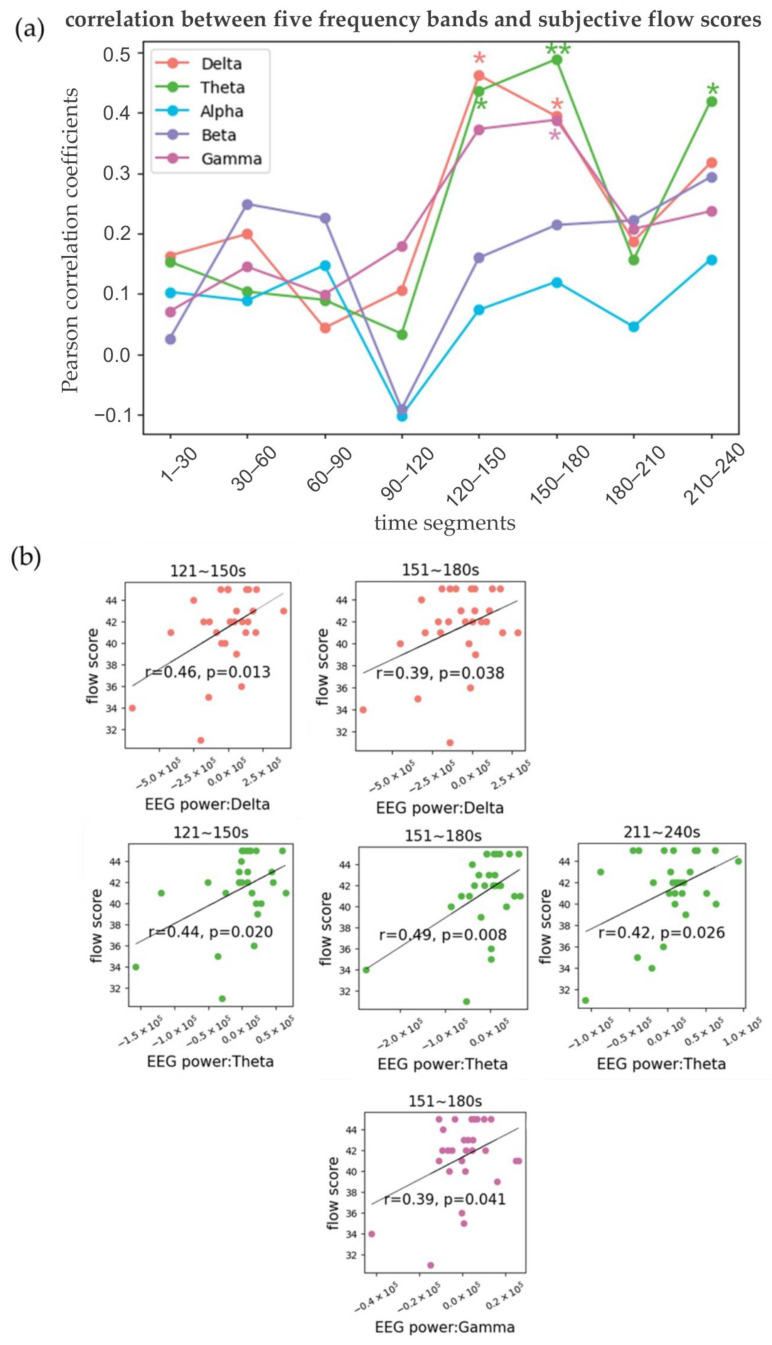
Correlation between the five frequency bands and the subjective flow scores in the 8 different segments. (**a**) Pearson correlation between power values of five frequency bands and subjective flow score (*: *p* < 0.05; **: *p* < 0.01, the same below). (**b**) Scatterplots in the specific time segments with significant correlations between specific frequency bands and subjective flow score.

In the formal EEG analysis, individual variations and the temporal dynamics of the flow experience have been taken into account. To this end, the initial 240 s of raw EEG data was processed, calculating the average every 30 s across the delta, theta, alpha, beta, and gamma frequency bands for each experimental condition, following baseline correction. The methodology involved selecting the most intense flow experience for each participant, characterized by the highest flow score, along with the corresponding EEG power. An analysis was conducted to compute the Pearson correlation between the EEG power and the subjective flow scores across each of the eight time segments and for each frequency band. The Pearson correlation analysis uncovered significant correlations between EEG power values in the delta, gamma, and theta frequency bands and subjective flow scores across various time segments, as shown in Figure 6 and Table 1. Specifically, for the delta frequency band, there was a positive correlation between EEG power values and flow scores in the segments from 121 to 150 s (*r* = 0.46, *p* = 0.013) and from 151 to 180 s (*r* = 0.39, *p* = 0.038), suggesting an association of higher delta band activity with enhanced flow experiences during these periods. Regarding the gamma frequency band, a positive correlation emerged in the segment from 151 to 180 s (*r* = 0.39, *p* = 0.041). This correlation indicates that periods of reported increased flow are associated with heightened gamma band activity, which may reflect more intense cognitive processing or engagement. For the theta frequency band, significant positive correlations with flow scores were evident in three time segments: from 121 to 150 s (*r* = 0.44, *p* = 0.020), from 151 to 180 s (*r* = 0.49, *p* = 0.008), and from 211 to 240 s (*r* = 0.42, *p* = 0.026). These results imply a steady link between theta band activity and the experience of flow throughout these segments.

### 3.3. Predictive Modeling of Flow Experiences

**Figure 7:**
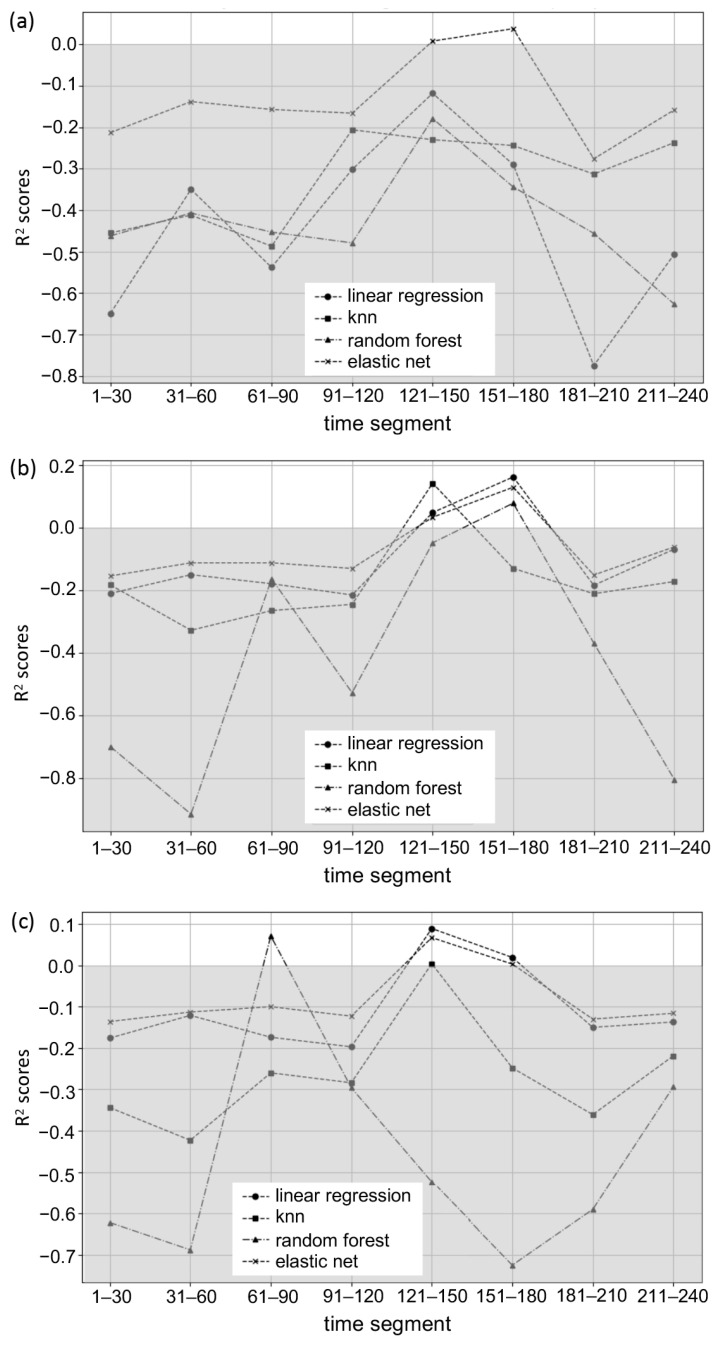
The R^2^ of different models across eight time segments, with three bands showing significant positive R^2^ scores: (**a**) combinations of five frequency bands, (**b**) theta band, and (**c**) delta band (areas in grey represent predictive models corresponding to cross-validation regressions that performed worse than simply guessing the mean).

In the final analysis, leave-one-subject-out cross-validation regressions were conducted to assess EEG power’s ability to predict the subjective flow score, considering both the combined effect of all five frequency bands (delta, theta, alpha, beta, gamma) and their individual contributions. A variety of models, such as KNN, elastic net, random forest, and linear regression, were utilized across different time segments to determine R^2^ for these time segments under each model. The combination of all five frequency bands showed a positive R^2^ of 0.008 under the elastic net model in time segment 5 (121~150 s), and a positive R^2^ of 0.038 under the same model in time segment 6 (151~180 s) (Figure 7a).

The theta band exhibited positive R^2^ values of 0.034, 0.143, and 0.049 under the elastic net, KNN, and linear regression models, respectively, in time segment 5 (121~150 s) and demonstrated positive R^2^ values of 0.13, 0.163, and 0.079 under the elastic net, linear regression, and random forest models, respectively, in time segment 6 (151~180 s) (Figure 7b). For the delta band, positive R^2^ values of 0.067, 0.004, and 0.089 were recorded under the elastic net, KNN, and linear regression models, respectively, in time segment 5 (121~150 s). Positive R^2^ values of 0.003 and 0.019 were noted under the elastic net and linear regression models, respectively, in time segment 6 (151~180 s) (Figure 7c).

## 4. Discussion

This study first investigated the induced psychological flow through the Tetris game, revealing insights into the relationship between objective difficulty levels and subjective flow experiences. The findings indicate that subjective flow experience cannot be solely determined by the objective difficulty level of an activity. This deviation from previous research emphasizes the importance of considering subjective scores in flow experiences.

The analysis of the best flow experiences across six different activities, as reflected in self-reported flow ratings, revealed significant individual variability in the activities that most effectively induce flow. The finding suggests a general trend towards artistic activities in eliciting strong flow experiences at the group level. However, the paradigm that induces the highest flow still differs from person to person. This variability in flow induction can be attributed to individual differences in interests, skills, and psychological or physiological responses to specific tasks. It also suggests that a one-size-fits-all approach to designing activities for flow induction may not be effective and underscores the need for employing multiple paradigms and personalizing activities to understand flow experiences.

Correlation analysis between EEG and behavior data showed that delta, gamma, and theta values positively correlated with flow scores significantly during various time segments. First, in the study, a significant positive correlation was observed between EEG power values in the delta frequency band and subjective flow scores. The results are similar to a study on professional tightrope performance [51], which found higher delta oscillation in the flow condition compared to the stress condition. The functional delta oscillations play a crucial role in synchronizing brain activity with autonomic functions and are key players in various motivational processes related to reward mechanisms, primal defensive responses, and heightened emotional engagement. Knyazev’s research [52,53] further emphasizes the integral role of delta oscillations in cognitive processes, especially those associated with attention and the identification of motivationally significant stimuli. In the context of heightened flow states, characterized by deep absorption and focused motivation toward task goals or rewards, there is an observed increase in delta activity. This heightened delta activity suggests not only a profound emotional connection but also a strong motivation driving individuals during flow experiences. It goes beyond mere absorption in the task, indicating an emotional involvement that contributes significantly to the flow phenomenon. Harmony [54] delves into this aspect by reviewing relevant research and proposing an additional layer to the understanding of sustained delta oscillations. According to Harmony [54], these sustained delta oscillations serve to prevent disruptions that could impact the execution of cognitive tasks. This prevention mechanism operates by potentially regulating the activity of networks that should remain inactive to accomplish the task at hand. Therefore, the increased delta activity observed during high flow states is not only indicative of emotional engagement and focused motivation but also aligns with Harmony [54]’s proposition. This sustained delta activity acts as a safeguard, tuning out irrelevant sensory information and facilitating a deep level of concentration. This intricate mechanism aligns seamlessly with the profound focus and absorption characteristic of flow states, providing a comprehensive perspective on the role of delta oscillations in the cognitive experience.

Second, a positive correlation was also observed within the gamma band. This result contradicts previous research [55] examining collective flow in a music rhythm game in which flow conditions demonstrated lower beta and gamma power in the prefrontal cortex (PFC) compared to no-flow conditions. Our research presents a seemingly contradictory picture to prior findings, possibly due to differing experimental paradigms. As found, gamma waves are frequently linked to advanced cognitive functions, with research such as Jensen [56]’s study demonstrating their connection to attention and working memory. This discrepancy may be attributed to the specific nature of the cognitive tasks, or the experimental settings employed in different studies. For instance, while studies like the one on collective flow in a music rhythm game observed lower gamma power in flow states, these findings might be specific to the social and interactive nature of the task. In contrast, our study, focusing on individual cognitive tasks, might engage different neural mechanisms, thereby eliciting an increase in gamma activity associated with flow. The positive correlation observed supports the notion that gamma waves are integral to high-level cognitive functions, as suggested by Jensen [56] and others. This is in line with the understanding that gamma activity facilitates complex information processing and neural synchronization, essential for maintaining a state of deep, focused engagement characteristic of flow. Furthermore, the increase in gamma activity could be reflecting the heightened attention, working memory, and sensory integration required during flow states.

Third, a distinct trend was observed within the theta frequency band. Significant positive correlations between theta activity and flow scores were identified at specific time segments. These findings align with observations made by Katahira [6] regarding elevated theta activities during mental arithmetic tasks in flow and cognitive overload conditions, compared to boredom. Previous studies underscore the importance of frontal theta activity in cognitive control [57]. For example, Ishii et al. [58] demonstrated a link between increased frontal theta activity and improved attention and processing efficiency, especially during mentally demanding tasks such as calculations. Our research reveals a dynamic interplay between theta-band activity and the subjective experience of flow, indicating that the flow experience involves higher cognitive control. The alignment of increased theta activity with the subjective experience of flow during these time segments suggests that theta rhythms may serve as neural markers of deep cognitive engagement.

Our study’s exploration of the relationship between EEG indicators and subjective flow experiences presents novel insights, particularly regarding the temporal dynamics of flow. The correlation between EEG measurements and subjective flow experiences becomes increasingly pronounced in the middle to later stages of the flow experience, especially in the 151–180 s time segment. During this period, a significant positive correlation was observed between delta, gamma, and theta values and subjective flow scores. This finding suggests that EEG indicators possess considerable time specificity in predicting the flow experience. One key observation is that when subjects initially enter the flow state, EEG features may not immediately reflect the subjective flow experience. This could be attributed to the fact that the subjects reported their flow scores post-experimentally, which might not accurately capture their subjective flow state at the onset of the experience. Our results, although not directly comparable with existing literature due to the innovative use of EEG in this context, intuitively align with the expectation that significant flow expression requires a certain duration to manifest. It is important to acknowledge that the specific time window identified for significant flow expression in our study may be unique to the tasks employed and the experimental setup. These results, while pioneering in demonstrating how flow unfolds over time using EEG, await further validation and exploration in future research. Subsequent studies should aim to corroborate our findings, thereby contributing to a more comprehensive understanding of the temporal aspects of flow experiences and the utility of EEG in capturing these dynamics.

In terms of the implications of our regression analysis, this study focused on employing EEG frequency bands as predictors for subjective flow scores. This analysis utilized a variety of models, including KNN, elastic net, random forest, and linear regression, applied across distinct temporal segments. R^2^ was calculated for each time segment under these models. The results of predictive modeling conducted for five separate frequency bands are largely consistent with the correlation findings. Specifically, both delta and theta bands exhibited R^2^ values greater than 0 in the fifth and sixth time segments (121~150 s and 151~180 s), aligning with significant correlations between EEG power and flow scores in these segments for delta and theta. However, theta did not show a positive R^2^ value in the eighth time segment (211~240 s), nor did gamma in the sixth segment (151~180 s), which diverges from the correlation results. The analysis through predictive modeling further identified more generalizable and robust features, specifically delta and theta in the fifth-(121~150 s) and sixth-(151~180 s) time segments. On the other hand, the overall results of predictive modeling for the combined five frequency bands were not as strong, possibly due to the limited number of participants and the complexity of the information. The relatively weaker performance of KNN and random forest in predictive modeling may also be attributed to these methods’ greater demand for data volume. These varying R^2^ values across models and time segments reflect the complex and dynamic nature of the neural correlates of flow. The predictive modeling approach employed here opens the possibility for practical applications, particularly in the development of brain–computer interfaces (BCIs) that can measure psychological flow. Such interfaces could have far-reaching implications for enhancing performance and well-being in various settings, such as in education, workplace environments, and therapeutic contexts. The differing predictive powers of the models at various time segments also highlight the importance of considering the temporal dynamics of flow in the design of such technologies, and future research could explore the integration of these models into practical applications. By leveraging the distinctive temporal patterns revealed in our analysis, BCIs could be tailored to detect the onset and progression of flow states, providing real-time feedback and interventions to maintain or enhance these states. This could lead to more personalized and effective approaches in areas where flow is critical to performance and satisfaction.

The research highlights two key aspects. First is the individual variability in flow experiences. This study addresses the individual variability of flow experiences by selecting and analyzing data from tasks that most effectively induce flow in each participant. Such an approach minimizes the impact of individual differences on the accuracy of correlating EEG indicators with subjective flow scores. This individualized methodology is crucial, as it acknowledges that flow is a highly subjective experience, varying significantly from person to person. Second is the temporal dynamics of EEG correlations. The study also focuses on the temporal dynamics between EEG indicators and behavioral measures of flow. A general upward trend in correlations across different frequency bands is observed. However, significant positive correlations are predominantly noted in specific bands during the middle and late stages of the flow experience. This temporal aspect is vital as it suggests that the brain’s response in flow states evolves over time and is most pronounced during certain periods.

The results of regression analysis in this study point to promising future applications in the realm of BCIs. Admittedly, the regression results (R^2^ of around 0.15) do not appear to be exceptionally high in magnitude. However, these results are comparable with the previous studies conducted in naturalistic settings using similar wearable devices [41,59], as well as some of the previous application-oriented BCI studies [60,61,62]. As relatively simple regression methods were employed, the results are expected to further be improved with advanced machine learning methods. For instance, numerous studies have used deep learning techniques for physiological emotion computation, notably using models like convolutional neural network(CNN), long short-term memory network, and attention-based convolutional recurrent neural network [63,64,65,66]. Specifically, cross-subject EEG emotion recognition [67], and in the context of this paper, the challenging topic of cross-subject flow prediction could involve innovative algorithms such as the Contrastive Learning method for Inter-Subject Alignment (CLISA) for cross-subject psychological flow level prediction [68], potentially enhancing flow research applications.

In conclusion, this study has illustrated the feasibility of using a lightweight, portable EEG device for objective, personalized flow measurements in natural task settings, contributing significantly to the field of neuroscientific research on psychological flow. It also underscores the potential of portable EEG technology in capturing the complex, dynamic nature of flow experiences, opening new avenues for practical applications.

## Figures and Tables

**Table 1:** Pearson correlation coefficients between five frequency bands and subjective flow scores in 8 different time segments: *r* value (*p*-value). *: *p* < 0.05; **: *p* < 0.01.

	Delta	Theta	Alpha	Beta	Gamma
1~30 s	0.16 (0.407)	0.15 (0.436)	0.10 (0.602)	0.03 (0.897)	0.07 (0.720)
31~60 s	0.20 (0.309)	0.10 (0.600)	0.09 (0.654)	0.25 (0.202)	0.15 (0.462)
61~90 s	0.04 (0.826)	0.09 (0.649)	0.15 (0.454)	0.23 (0.249)	0.10 (0.616)
91~120 s	0.11 (0.590)	0.03 (0.865)	−0.10 (0.606)	−0.09 (0.645)	0.18 (0.362)
121~150 s	0.46 * (0.013)	0.44 * (0.020)	0.07 (0.711)	0.16 (0.417)	0.37 (0.050)
151~180 s	0.39 * (0.038)	0.49 ** (0.008)	0.12 (0.543)	0.21 (0.274)	0.39 * (0.041)
181~210 s	0.19 (0.341)	0.16 (0.426)	0.05 (0.818)	0.22 (0.257)	0.21 (0.289)
211~240 s	0.32 (0.100)	0.42 * (0.026)	0.16 (0.424)	0.29 (0.129)	0.24 (0.225)

## Data Availability

The data used to support the findings of this study are available from the corresponding author upon request.
